# Association between physical activity and longitudinal change in body mass index in middle-aged and older adults

**DOI:** 10.1186/s12889-023-15119-7

**Published:** 2023-01-30

**Authors:** Laura Cleven, Jeremy A. Syrjanen, Yonas E. Geda, Luke R. Christenson, Ronald C. Petersen, Maria Vassilaki, Alexander Woll, Janina Krell-Roesch

**Affiliations:** 1 Institute of Sports and Sports Science, Karlsruhe Institute of Technology, Engler-Bunte-Ring 15, 76131 Karlsruhe, Germany; 2 Department of Quantitative Health Sciences, Mayo Clinic, Rochester, MN USA; 3 Department of Neurology and the Franke Global Neuroscience Education Center, Barrow Neurological Institute, Phoenix, AZ USA; 4 Department of Neurology, Mayo Clinic, Rochester, MN USA

**Keywords:** Trajectories, BMI, Activity, Exercise, Community, Longitudinal, Adults

## Abstract

**Background:**

In middle-aged and particularly older adults, body mass index (BMI) is associated with various health outcomes. We examined associations between physical activity (PA) and longitudinal BMI change in persons aged ≥ 50 years.

**Methods:**

The sample included 5159 community-dwelling individuals aged ≥ 50 years (50.5% males, mean (SD) age 73.0 (10.2) years at baseline) who were enrolled in the Mayo Clinic Study of Aging (MCSA). Participants had information on PA within one year of baseline assessment, BMI at baseline, and potential follow-up assessments (mean (SD) follow-up 4.6 (3.7) years). Linear mixed-effect models were used to calculate the association between PA (moderate-vigorous physical activity, MVPA; and all PA composite score) and the longitudinal change in BMI, adjusted for baseline age, sex, education and medical comorbidities. In addition to interactions between years since baseline and PA, we also included 2- and 3-way interactions with baseline age to further assess whether age modifies the trajectory of BMI over time.

**Results:**

We observed a decrease in BMI among participants engaging at a mean amount of PA (i.e., MVPA: 2.7; all PA: 6.8) and with a mean age (i.e., 73 years) at baseline (MVPA: estimate = -0.047, 95% CI -0.059, -0.034; all PA: estimate = -0.047, 95% CI -0.060, -0.035), and this decline is accelerated with increasing age. Participants with a mean age (i.e., 73 years) that engage at an increased amount of MVPA or all PA at baseline (i.e., one SD above the mean) do not decrease as fast with regard to BMI (MVPA: estimate = -0.006; all PA: estimate = -0.016), and higher levels of MVPA or all PA at baseline (i.e., two SD above the mean) were even associated with an increase in BMI (MVPA: estimate = 0.035; all PA: estimate = 0.015). Finally, MVPA but not all PA is beneficial at slowing BMI decline with increasing age.

**Conclusion:**

PA, particularly at moderate-vigorous intensity, is associated with slower decline in longitudinal BMI trajectories. This implies that engaging in PA may be beneficial for healthy body weight regulation in middle and late adulthood.

## Introduction

The prevalence of overweight and obesity in adults has been increasing over past decades [1]. A growing body of research has demonstrated relationships between higher body weight or body mass index (BMI) and all-cause mortality [2], as well as various medical conditions [3]. While human body weight regulation is complex, engaging in physical activity (PA) is generally associated with reduced body weight or fat in young and middle-aged adults (e.g., [4]). Similarly, longitudinal studies in adults have demonstrated associations between PA and reduced risk of onset of several metabolic diseases including overweight and obesity (e.g., [5]), dementia [6, 7] as well as all-cause mortality [8].

In contrast, among older adults, weight loss, particularly at rapid decline, may be associated with undesirable health outcomes. For example, a more pronounced decline in weight or BMI per decade from mid- to late-life was associated with an increased risk of incident mild cognitive impairment [9]. Similarly, greater BMI instability was associated with faster cognitive decline over an average follow-up of 6 years [10]. However, conflicting results on the association between BMI and cognitive decline have been reported, e.g. studies have shown that a higher BMI may be associated with an increased risk of dementia in longitudinal follow-up (i.e., when BMI was assessed > 20 years before dementia diagnosis), but with a decreased risk of dementia in relatively shorter follow-up (i.e., when BMI was assessed < 10 years before dementia diagnosis [11]). Other studies have also reported associations between a lower BMI and increased risk of mortality [12] or disability in activities of daily living [13] among older community-dwelling persons.

Few studies investigated the associations between PA and BMI in older adults, and results are inconsistent. For example, studies reported an increase in impairments of activities of daily living for persons aged ≥ 65 years with obesity compared to those with low or normal body weight [14], as well as an association between lower levels of PA and physical function in obese individuals aged ≥ 60 years [15]. However, another study reported lower odds of physical and functional impairment when engaging in moderate to vigorous intensity exercise at least once per week in a sample of obese older adults [16].

The aim of this study was to examine the longitudinal associations between PA and BMI trajectories in community-dwelling persons aged ≥ 50 years. Based on previous literature, marked BMI instability in old age is associated with undesirable health outcomes. It is therefore critical to investigate whether engagement in PA is associated with change in BMI over time. These findings may be relevant for the promotion of a healthy lifestyle in clinical practice, particularly among older persons.

## Methods

### Study design and sample

This prospective study was conducted in the setting of the population-based Mayo Clinic Study of Aging (MCSA) in Olmsted County, Minnesota, USA [17]. We included 5159 individuals aged ≥ 50 years with available data on PA within 12 months of baseline assessment, and BMI at baseline and potential follow-up assessments. Participants were followed forward in time for a mean of 4.6 years. The MCSA protocols have been approved by the institutional review boards (IRB) of Mayo Clinic and Olmsted Medical Center in Rochester, MN, USA. All participants provided written informed consent before participation. In the case of participants with cognitive impairment sufficient to interfere with capacity, consent was obtained from a legally authorized representative.

### Measurement of predictor variables

PA was measured at baseline using a self-reported questionnaire [18–20], derived from two validated instruments, i.e., the 1985 National Health Interview Survey and the Minnesota Heart Survey intensity codes [21, 22]. This questionnaire has been used in previous studies on PA in the context of aging and cognitive impairment [19, 20]. Details of the survey was reported elsewhere [16]; briefly, the questionnaire inquired about engagement in PA as well as exercise in late-life (i.e., over the past one year) and distinguished between three intensity levels by providing examples for each level: (1) light PA (such as laundry, vacuuming, making beds or dusting); (2) moderate PA (such as scrubbing floors, washing windows, gardening or raking leaves); (3) heavy PA (such as carrying heavy objects, heavy digging, pushing a mower or hard manual labor); (4) light physical exercise (such as leisurely walking or slow dancing); (5) moderate physical exercise (such as hiking or swimming); and (6) vigorous physical exercise (such as jogging or playing tennis singles). Participants were asked to provide information about the frequency at which they carried out these activities: ≤ 1 time per month, 2–3 times per month, 1–2 times per week, 3–4 times per week, 5–6 times per week, and daily. For the purpose of this study, we calculated two scores: (1) A late-life all PA composite score by taking light, moderate, and vigorous physical activities and exercise, converting each to days per week, adding them up and dividing the total by 2 to get an overall score. The scores range from 0 to 21, with a higher score indicating higher level of PA. (2) A late-life moderate-vigorous physical activity (MVPA) score by adding the days per week variables for moderate and vigorous exercise. Both scores were z- scored for use in the models.

### Measurement of outcome variables

Participants’ weight (in kg) and height (in m) was measured at each study visit to calculate BMI according to the formula: kg/m^2^. For descriptive purpose, we categorized participants into six BMI groups [23]: <18.5 (underweight), ≥ 18.5 to < 25 (normal/ healthy weight), ≥ 25 to < 30 (overweight), ≥ 30 to < 35 (obesity class I), ≥ 35 to < 40 (obesity class II), ≥ 40 (obesity class III). BMI was measured every 15 months on average.

### Covariates

We assessed age, sex, years of education based on ten different educational level determinations (e.g., high school diploma = 12 years, Bachelor’s degree = 16 years, Master’s degree = 18 years, etc.), and medical comorbidity as assessed through the weighted Charlson Index [24].

### Statistical analysis

**Fig. 1:**
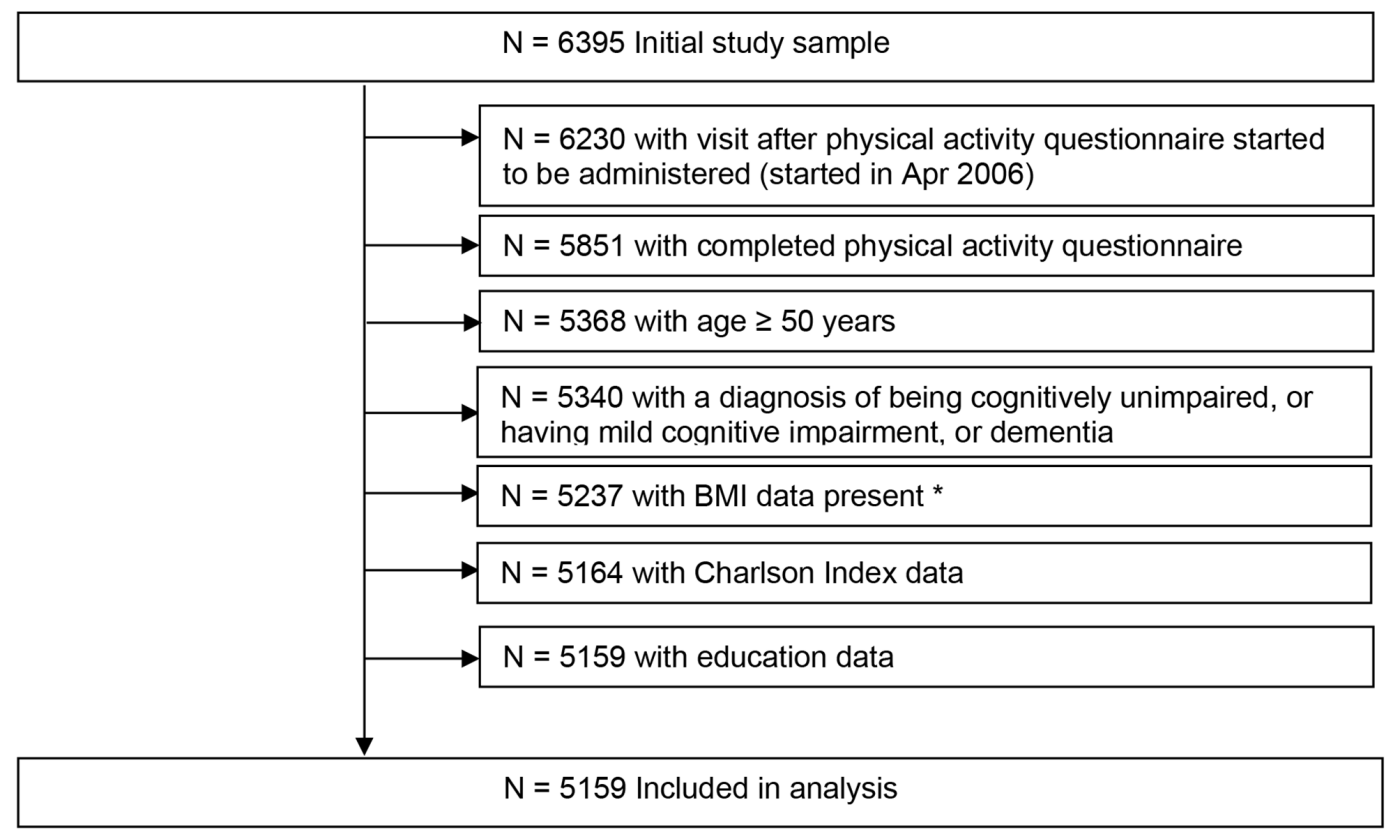
Flow chart *BMI was included even if data was only available at baseline visit but no follow-up Abbreviations: BMI = body mass index

**Fig. 2:**
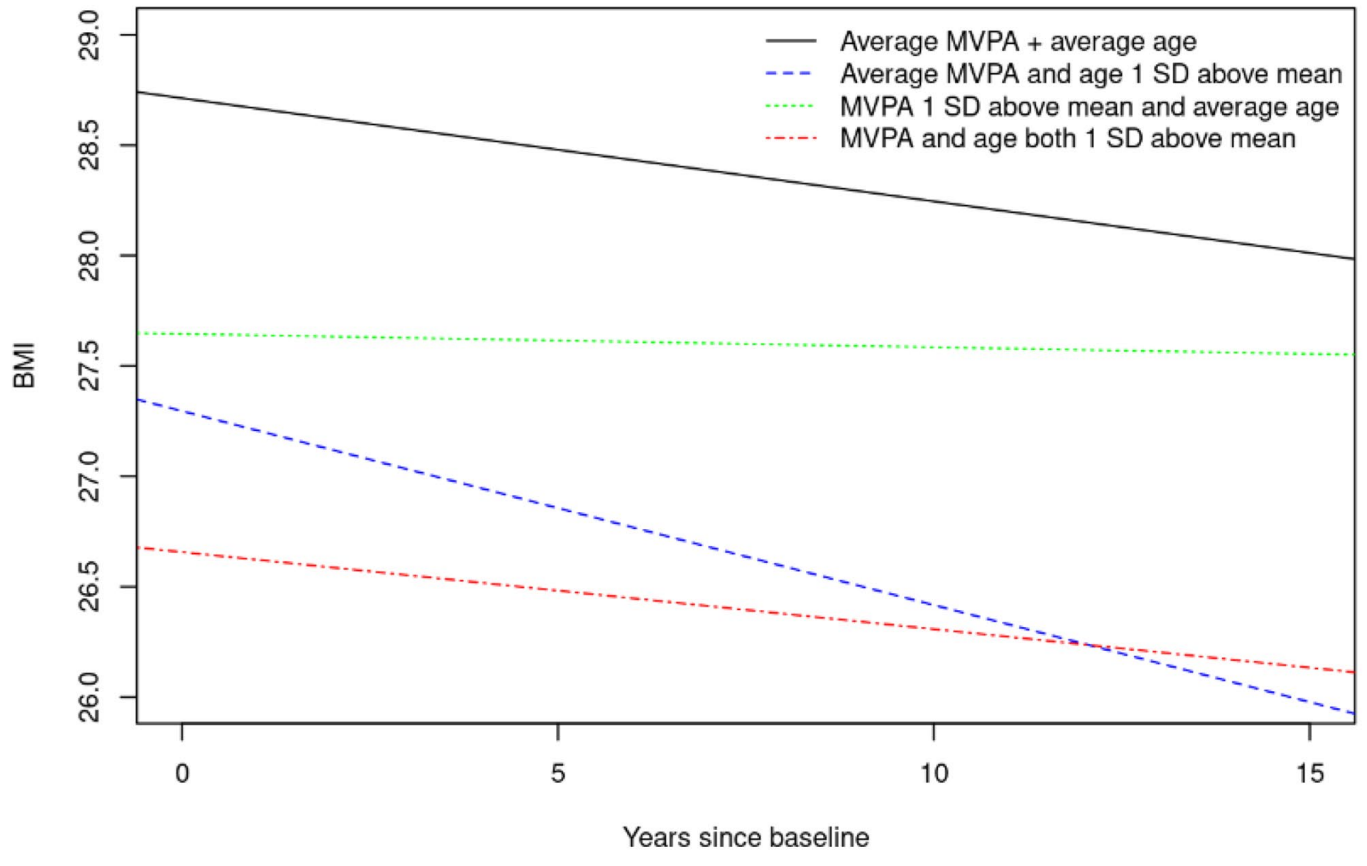
Change in BMI over time given age and level of MVPA (last 12 months) Abbreviations: MVPA = moderate-vigorous physical activity; SD = standard deviation

**Fig. 3:**
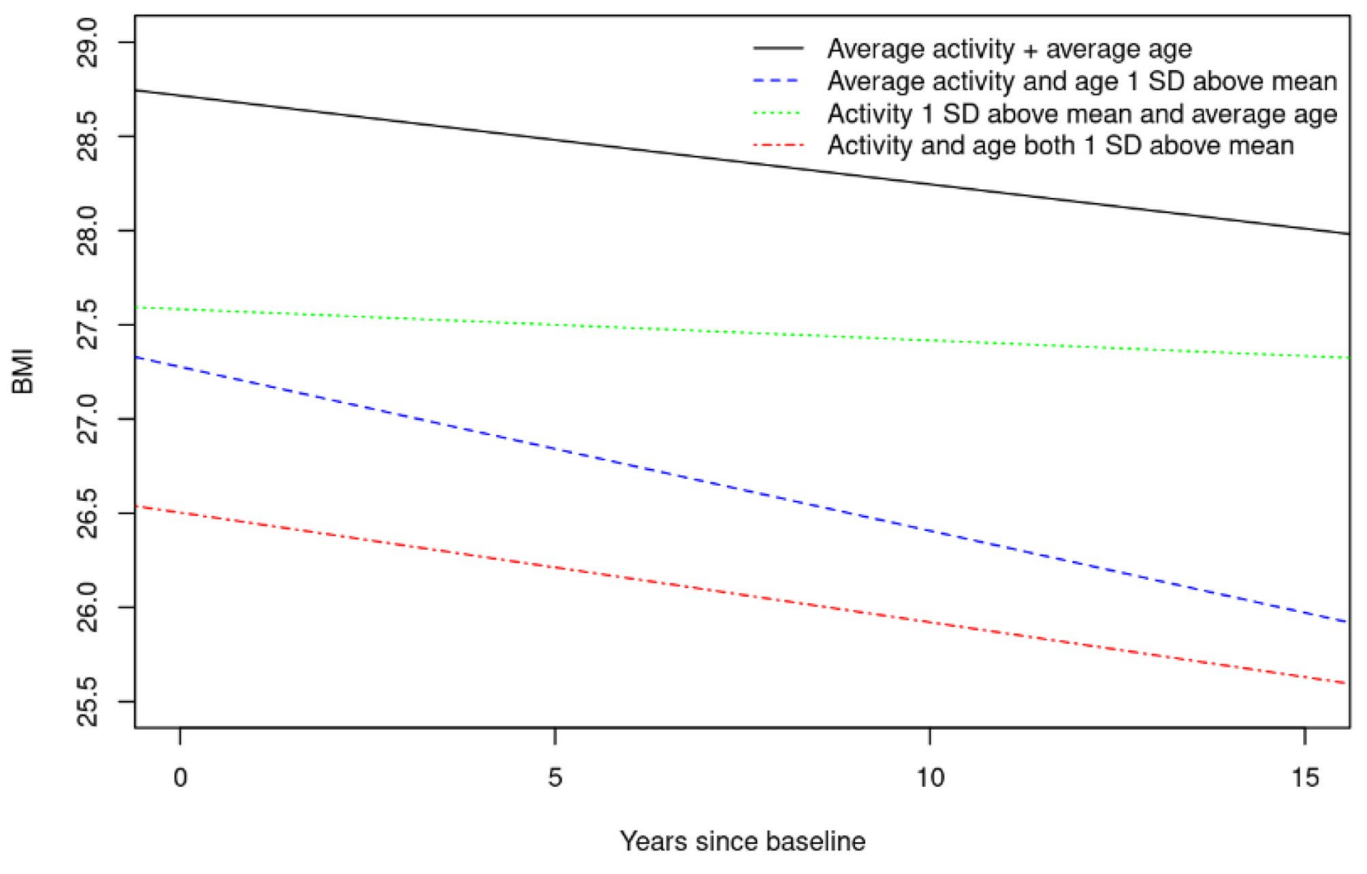
Change in BMI over time given age and level of all PA (last 12 months) Abbreviations: SD = standard deviation

We calculated linear mixed-effect models with random subject-specific intercepts and slopes to examine the association between late-life PA at baseline and change in BMI over time. In our models, the PA variables were the independent variables (predictors), and the trajectories for individual yearly longitudinal change in BMI were the dependent variables (outcomes). We only included participants who had information on PA, years of education, and Charlson Index at baseline, respectively; were aged ≥ 50 years; and had information on BMI at baseline and potentially follow-up assessments. For a detailed flow chart on the sample selection, please refer to Fig. 1. All models included PA (z-scored), sex, time in years from baseline, age (z-scored), education years and Charlson comorbidity index and 2- and 3-way interactions amongst PA, age, and time in years since baseline. We conducted the analyses separately for all PA (z-scored composite score) and z-scored MVPA score. To help visualize the results, given that 2- and 3-way interactions can be difficult to interpret, we created plots (please refer to Fig. 2 and Fig. 3) utilizing the fixed effects equations from our models. In each plot are four lines showing BMI trajectory over time for (1) those with average PA and average age, (2) those with average PA and age one standard deviation (SD) above the mean, (3) those with PA one SD above the mean and average age, and (4) those with both PA and age one SD above the mean, respectively. All covariates were considered to have the average value for the purposes of the plots. All statistical analyses were done using the conventional two-tailed alpha level of 0.05 and performed with SAS 9.4 (SAS Institute, Inc; Cary, NC) and R, version 4.1.2 (R Foundation for Statistical Computing, Vienna, Austria).

## Results

Detailed descriptive demographics at baseline are shown in Table 1. In our sample of 5159 participants, the mean (SD) age was 73.0 (10.2) years, and the mean (SD) BMI at baseline was 28.6 (5.6). The mean (SD) follow-up was 4.6 (3.7) years. 914 participants had one assessment of BMI, 812 participants had two assessments, 640 participants had three assessments, 573 had four assessments, and 2220 participants had ≥ five assessments of BMI. The mean (SD) times of follow-up BMI assessments was of 4.4 (2.8).

**Table 1:** Characteristics of participants at baseline

	Total (N = 5159)
Age in years, Mean (SD)	73.0 (10.2)
Age categories, N (%)	
50–59 years	682 (13.2)
60–69 years	1116 (21.6)
70–79 years	1898 (36.8)
80–89 years	1378 (26.7)
≥ 90 years	85 (1.6)
Male sex, N (%)	2605 (50.5)
Education in years, Mean (SD)	14.4 (2.7)
Cognitive Status, N (%)	
Normal	4503 (87.3)
Mild cognitive impairment	588 (11.4)
Dementia	68 (1.3)
Late-life PA composite score, Mean (SD)	6.8 (3.9)
Late-life MVPA, Mean (SD)	2.7 (3.0)
BMI, Mean (SD)	28.6 (5.6)
BMI group, N (%)	
<18.5	44 (0.9)
≥ 18.5 to < 25	1302 (25.2)
≥ 25 to < 30	2071 (40.1)
≥ 30 to < 35	1140 (22.0)
≥ 35 to < 40	396 (7.7)
≥ 40	206 (4.0)
Charlson index, Mean (SD)	3.4 (3.3)
Smoker, N (%)	2377 (46.1)
Marital status, N (%) ^{1}^	
Single, never married	238 (4.6%)
Married	3588 (69.6%)
Separated	12 (0.2%)
Divorced	424 (8.2%)
Widowed	865 (16.8%)
Living together, not married	30 (0.6%)
Other, specify	1 (0.0%)

Abbreviations: N = number of participants; SD = standard deviation; PA = physical activity; MVPA = moderate-vigorous physical activity; BMI = body mass index. Age range 50.0-94.2 years; years of education range 5–20 years; late-life physical activity composite score range 0–21; late-life MVPA score range 0.2–14; BMI range 15.0-62.9; Charlson index range 0–22; smoking was assessed using the item: ‘Do you smoke cigarettes now? yes - no’; $\underline{\underline {\{ \} }} =$ indicates number of persons with missing data

### Association of MVPA (last 12 months) and change in BMI

Participants engaging at an average amount of MVPA over the past 12 months and at the average age at baseline, decrease in BMI annually (estimate = -0.047, 95% CI [-0.059, -0.034], p < 0.0001) (Table 2). Those that are at the average for MVPA but are older at baseline (i.e., someone who is one SD above the mean age), fall even faster in terms of their BMI (-0.047–0.041 = -0.088). On the other hand, participants at the average age that perform an increased amount of MVPA (i.e., someone who is one SD above the mean MVPA) do not decrease as fast on BMI (-0.047 + 0.041 = -0.006), and at higher levels of MVPA (i.e., someone who is two SD above the mean in MVPA), BMI starts to increase over time (-0.047 + 2*0.041 = 0.035). There was also a marginally significant positive (estimate = 0.012, 95% CI [-0.001, 0.026], p = 0.068) 3-way interaction between years since baseline, age at baseline, and MVPA. This means that, the older one gets, the more beneficial MVPA is at mitigating BMI decline, as displayed in Fig. 2.

**Table 2:** Association of MVPA (last 12 months; independent variable) and change in BMI (dependent variable)

Effect	Estimate	95% CI	p
Intercept	30.273	29.455, 31.092	< 0.0001
Male sex	0.674	0.382, 0.966	< 0.0001
Education years	-0.174	-0.228, -0.119	< 0.0001
Charlson index	0.180	0.132, 0.228	< 0.0001
Years since baseline	-0.047	-0.059, -0.034	< 0.0001
Age	-1.417	-1.575, -1.259	< 0.0001
MVPA last 12 months	-1.068	-1.216, -0.919	< 0.0001
Years since baseline * Age	-0.041	-0.055, -0.028	< 0.0001
Years since baseline * MVPA last 12 months	0.041	0.028, 0.0532	< 0.0001
Age * MVPA last 12 months	0.429	0.284, 0.573	< 0.0001
Years since baseline * Age * MVPA last 12 months	0.012	-0.001, 0.026	0.068

Abbreviations: 95% CI = 95% confidence interval; MVPA = moderate-vigorous physical activity. MVPA and age were z-scored

### Association of all PA (last 12 months) and change in BMI

Participants engaging at an average amount of all PA over the past 12 months and at the average age at baseline, decrease in BMI annually (estimate = -0.047, 95% CI [-0.060, -0.035], p < 0.0001) (Table 3). Those that are at the average for all PA but are older at baseline (i.e., someone who is one SD above the mean in age), fall even faster in terms of their BMI (-0.047 – 0.040 = -0.087). On the other hand, participants at the average age that perform an increased amount of all PA (i.e., someone who is one SD above the mean all PA) do not decrease as fast on BMI (-0.047 + 0.031 = -0.016), and at higher levels of all PA (i.e., someone who is two SD above the mean in all PA), BMI starts to increase over time (-0.047 + 2*0.031 = 0.015). We did not observe a 3-way interaction (estimate = -0.002, 95% CI [-0.015, 0.012], p = 0.806) between years since baseline, age at baseline, and all PA, as displayed in Fig. 3.

**Table 3:** Association of all PA (last 12 months; independent variable) and change in BMI (dependent variable)

Effect	Estimate	95% CI	p
Intercept	30.629	29.813, 31.444	< 0.0001
Male sex	0.649	0.358, 0.940	< 0.0001
Education years	-0.193	-0.248, -0.139	< 0.0001
Charlson index	0.163	0.115, 0.211	< 0.0001
Years since baseline	-0.047	-0.060, -0.035	< 0.0001
Age	-1.440	-1.598, -1.281	< 0.0001
All PA last 12 months	-1.133	-1.282, -0.985	< 0.0001
Years since baseline * Age	-0.040	-0.053, -0.026	< 0.0001
Years since baseline * All PA last 12 months	0.031	0.018, 0.043	< 0.0001
Age * All PA last 12 months	0.360	0.215, 0.505	< 0.0001
Years since baseline * Age * All PA last 12 months	-0.002	-0.015, 0.012	0.806

Abbreviations: PA = physical activity; 95% CI = 95% confidence interval. All physical activity and age were z-scored

## Discussion

Among participants engaging at a mean amount of PA (i.e., MVPA score of 2.7, or all PA score of 6.8) and with a mean age at baseline (i.e., 73 years), we observed a decrease in BMI over time. This decline is accelerated with increasing age. In addition, participants with a mean age (i.e., 73 years) that engaged at an increased amount of MVPA or all PA at baseline did not decrease as fast with regard to BMI, and higher levels of MVPA or all PA were even associated with an increase in BMI. Finally, MVPA but not all PA, is more beneficial at slowing BMI decline with increasing age. This result indicates that particularly PA engagement at higher intensities rather than overall PA may be important to slow a decline of BMI that may negatively impact health.

Previous longitudinal research has reported conflicting results. For example, studies have demonstrated a stable or decreasing BMI over time [25, 26], whereas others found an increase in BMI over time, albeit with differing trajectories for specific age groups (i.e., up to old age [25–28]). Results from longitudinal studies on the association between baseline levels of PA and BMI changes over time focusing on older adults are scarce, while those on middle-aged adults are conflicting [29, 30]. For example, some studies reported a significant association between higher levels of PA at baseline and a decreasing BMI [31] or a reduced weight gain [32, 33]. Whereas, other studies did not find such an association [34, 35]. The conflicting findings may be due to different sample sizes with regard to age, sex distribution, recruitment, as well as different study methodology, particularly with regard to the assessment of PA. Therefore, our study expands on the existing body of literature by showing that particularly MVPA may be associated with slower BMI decline over time in older adults.

We did not examine potential mechanisms that may explain our observed association between PA and slower decline in BMI trajectories in older adults. However, it has been postulated in the literature that BMI is not exclusively influenced by PA. Other behavioral factors such as nutrition (e.g. [36]), longitudinal patterns of PA behavior, e.g. stability of PA engagement over the lifespan [37, 38] as well as metabolic mechanisms related to PA (e.g. [39]) or socio economic status (e.g. [40]) may impact body weight management. Furthermore, in adults, a high BMI is usually due to a high fat mass which is associated with negative effects on health and may lead to reduced quality of life due to functional impairment [41]. However, of note, weight loss in older adults is mostly characterized by decline in lean body mass (particularly muscle mass) and is associated with an increased mortality, whereas a decline in body fat mass is associated with a reduced mortality [42]. As the mean age of our sample was 73 years at baseline, a low BMI may actually indicate a reduced skeletal muscle mass, which in turn is related to a reduction of physical functioning and an increased risk of falls [43]. In addition, weight gain in older adults may be beneficial for maintaining cognitive functioning, whereas weight loss should be avoided; and this has been referred to in literature as the obesity paradox in cognition [44]. Indeed, several studies reported that a decline in BMI was associated with an increased risk of incident mild cognitive impairment [9], faster rates of cognitive decline [45], as well as decreased cognitive performance [46] or abilities [47]. Since cognitive status may have an impact on the association between PA and BMI trajectories, we additionally adjusted our models for cognitive status at baseline (i.e., being cognitively unimpaired, or having mild cognitive impairment or dementia). However, this additional adjustment did not alter our results and conclusions (data not shown). In general, future research should explore potential mechanism that may explain our observations that PA and MVPA are differently associated with longitudinal BMI depending on the age of participants. One may hypothesize that this difference is due to types of PA or PA stimulus, personal (e.g., health) conditions, body composition, or BMI limitations when relating it to PA.

The strengths of our study are the follow-up period of 4.6 years, and the large sample of more than 5000 community-dwelling older individuals. Major limitations pertain to the use of a self-reported questionnaire to assess PA which may be likely to induce recall bias. However, previous research in a different sample derived from the MCSA has shown that our PA questionnaire has moderate to good internal consistency, and test-retest correlation coefficients range between 0.33 for vigorous intensity activity and 0.50 for moderate intensity activity [18]. Furthermore, only 26% of participants were not overweight or obese at baseline, which may have limited our ability in the statistical analyses to capture meaningful changes in BMI. There is limited evidence of an association between greater amounts of PA and attenuated weight gain with the effect diminishing with increasing age [30]. Further longitudinal studies are needed, particularly to investigate the effects of increasing or decreasing PA over time on BMI trajectories, and also the impact of continued versus interrupted PA engagement over the lifespan on BMI trajectories. In addition, with regard to the outcome of interest (i.e. BMI trajectories), it would be interesting to examine whether the PA related change in BMI is due to loss of lean body mass (mainly muscle mass) or body fat mass, which may have different implications for health in older adults. Additionally, future studies should also consider dietary intake as a covariate.

## Conclusion

In conclusion, we found that PA, particularly at moderate-vigorous intensity, carried out within 12 months of baseline data acquisition, is associated with a slower decrease in BMI. Given that a decline in BMI in old age can have negative health consequences, our findings underline the importance of engaging in PA to limit unhealthy weight loss in old age, and of promoting a healthy lifestyle, including but not limited to regular PA, in clinical practice, particularly among older persons who are at risk of unhealthy weight loss.

## Data Availability

The datasets used and analyzed during the current study are available to qualified researchers from the Mayo Clinic Study of Aging upon reasonable request (https://www.mayo.edu/research/centers-programs/alzheimers-disease-research-center/data-requests).

## Abbreviations

BMI: Body mass index
kg: Kilograms
m: Meters
MCSA: Mayo Clinic Study of Aging
MVPA: Moderate-vigorous physical activity
N: Number of participants
PA: Physical activity
SD: Standard deviation
